# β-Elemene: Mechanistic Studies on Cancer Cell Interaction and Its Chemosensitization Effect

**DOI:** 10.3389/fphar.2017.00105

**Published:** 2017-03-09

**Authors:** Ziyu Jiang, Joe A. Jacob, Dinesh S. Loganathachetti, Prasannabalaji Nainangu, Baoan Chen

**Affiliations:** ^1^Department of Hematology and Oncology, Zhongda Hospital, School of Medicine, Southeast UniversityNanjing, China; ^2^Department of Oncology, Affiliated Hospital of Integrated Traditional Chinese and Western Medicine, Nanjing University of Chinese MedicineNanjing, China; ^3^Department of Marine Biotechnology, Bharathidasan UniversityTiruchirappalli, India; ^4^Department of Microbiology, Bharathidasan UniversityTiruchirappalli, India

**Keywords:** β-elemene, anticancer, apoptosis, cytotoxic, mechanism

## Abstract

Over the past decade, screening and identifying novel compounds for their biomedical applications has become an upcoming area of research. Identifying the molecular mechanisms of these compounds has become an integral part of anticancer research. β-elemene, a sesquiterpene, is renowned for its anticancer activity against a variety of cell lines. Recent studies on β-elemene have elucidated that it possesses anti-proliferative effect on cancer cells by creating an apoptotic trigger. Interestingly, it also induces protective autophagy in some cancerous cell lines and is less cytotoxic compared to other widely accepted chemotherapeutic agents. This provides an edge with the perception of limited toxicity to normal cells. This mini-review precisely focuses on the studies performed to identify the mechanism of anticancer activity of β-elemene against cancer cells of multiple origin. In accordance to the evaluation made by the studies mentioned, apoptosis has been identified to be most possible reason behind anticancer activity exerted by β-elemene against a variety of cancer cell lines. Cell cycle arrest and necrosis have been credited to be possible alternate mechanisms for the anticancer effect of β-elemene.

## Introduction

Cancer is the reason behind highest number of deaths across the world and contemporary measures have become the need of the hour (Siegel et al., 2016). β-elemene is a sesquiterpene, with a wide spectrum of antineoplastic activity, even against drug-resistant tumors and other complex malignancies (Liu et al., 2011). Among its three isomers (δ, α, β), β-elemene (referred to as elemene, hereafter), is the predominant component (Wu et al., 2009).

The most well studied mechanism by which anticancer agents induce cell death in cancer cells is by the induction of apoptosis (Frankfurt and Krishan, 2003). DNA fragmentation and activation of caspases are the major hallmarks of apoptosis (Saraste and Pulkki, 2000). Apoptosis has been strongly induced in cancerous cells by elemene (Guan et al., 2014). Elemene, a prominent component of traditional chinese medicine (TCM) regulates several molecular targets and exerts promising anticancer effects (Jiang et al., 2016). Although elemene can induce antitumor effects in cancerous cells of multiple origin, the mechanism of action still remains unclear (Zhu et al., 2011b). In the current mini-review, the mechanism of action of elemene was reviewed and analyzed.

## Elemene Mechanism of Action Against Cancer Cells of Multiple Origin

### Integumentary System

In B16F10 melanoma cells, the expression of VEGF was inhibited by elemene elucidating its antiangiogenic activity. Similar results were obtained in rat aortic ring and chicken embryo chorioallantoic membrane. In C57BL/6 melanoma mice, western blot and immunohistochemical analysis showed that CD34 expression was reduced, further evidenced by reduction in tumor volume and suppression of VEGF-mediated angiogenesis. This study therefore determines that the antineoplastic effect of elemene in cells of melanoma origin is by an antiangiogenic effect (Chen et al., 2011).

### Nervous System

Mitogen-activated protein kinase kinase-3 (MKK3) and -6 (MKK6), is a kinase enzyme which phosphorylates mitogen-activated protein kinase (MAPK). When activated through mutual compensation, it can mediate the antiglioblastoma effect of elemene. In the human U87 glioblastoma cell line, the upregulation of MKK3 and MKK6 can lead to cell cycle arrest at the G0/G1 phase thereby leading to an anticancer effect of elemene (Zhu et al., 2011b). Elemene, in a similar study, inhibited the proliferation of U87 cells through the Glia maturation factor β -dependent inactivation of the ERK1/2-Bcl-2/survivin pathway. It also chemosensitized the glioblastoma cells to temozolomide (Zhu et al., 2014). In C6 and U251 glioblastoma cells, elemene treatment resulted in p38 MAPK phosphorylation and cell-cycle arrest at the G0/G1 phase (Yao et al., 2008a,b). Activation of Glia maturation factor β mediates the antitumor effect of elemene in glioblastoma cell line U87 and this can increase the sensitivity of cancer cells to cisplatin (Zhu et al., 2011a). Therefore, the antiglioblastoma effect of elemene is mediated through the cell cycle arrest at G0/G1 phase, leading to MAPK phosphorylation. The p38 MAPK phosphorylation is activated by the upregulation of MKK3 and MKK6 (Son et al., 2010). The activation of p38 MAPK eventually leads to cellular apoptosis (Koul et al., 2013). Therefore, these studies are an evidence for killing of glioblastoma cells by elemene via an apoptotic trigger.

Supporting this view, elemene increased caspase-3/7/10 activity, up-regulated Bax expression, and down-regulated the Bcl-2, Bcl-XL, and of X-linked inhibitor of apoptosis expressions. This suggests the role of apoptosis in anticancer effect of elemene on brain tumor cell lines A172, CCF-STTG1, and U-87MG (Li et al., 2013b). Elemene induced cell cycle arrest at G0/G1 phase in U87 cells and elevated the expressions of caspases-3, -8, -9, Fas, FasL, and Bax. It downregulated the expressions of Bcl-2, indicating the induction of apoptosis in the cancer cells (Li et al., 2014). In glioma U251 and A172 cells, elemene induced apoptosis through upregulation of caspase-9, -3 and -7 expressions and downregulation of survivin gene expression (Zhang et al., 2012). Against the rat glioma cell C6 and human glioma cell SHG-44, elemene possessed antiproliferative effects through induction of apoptosis (Zhou et al., 2003). These studies prove that the antineoplastic effect of elemene against cancers of nervous system is by the induction of apoptosis.

### Respiratory System

In the human NSCLC cell lines H460 and A549, a Chk2-dependent mechanism adopted by elemene caused the G2-M arrest. Elemene induced caspase-3, -7, and -9 activities, decreased Bcl-2 expression, resulted in cytochrome c release and increased the levels of cleaved caspase-9 and poly (ADP-ribose) polymerase through a mitochondrial release of the cytochrome c-mediated apoptotic pathway (Wang et al., 2005). Elemene and etoposide phosphate in synergism can induce the expressions of Bax, p53, and p21, mediated by cleavage of PARP and the suppression of cyclin D1 in A549 NSCLC cells (Zhang et al., 2011). Elemene can restore the sensitivity of NSCLC cells (PC9 and H1299) to Gefitinib by upregulation of p21 expression (Zhao et al., 2011). p21 is a key arbitrator of p53 function, which can lead to apoptosis (Xia et al., 2011). Elemene can induce the expressions of Bax and phospho-Bcl-2 and decrease Bcl-2 and XIAP expressions in human NSCLC cell lines H460 and A549. It can also increase the cisplatin-induced expressions of caspase-3, -7, -9, and -10 activities and cleaved caspase-3, -9, and poly (ADP-ribose) polymerase levels thereby sensitizing the cells to cisplatin by mitochondria-mediated intrinsic apoptosis pathway (Li et al., 2009). Elemene has antiproliferative effects on human NSCLC PC9, H1299, H1650, A549, H358, and H1975 cells by ERK1/2- and AMPKα-mediated inhibition of transcription factor Sp1, followed by reduction in DNMT1 protein expression (Zhao et al., 2015).

Elemene in synergism with Docetaxel resulted in increased cytochrome c release from mitochondria, significant caspase-8 and -3 cleavage, and downregulation of Bcl-2 and Bcl-XL expressions in p53 mutant H23 cells and p53 null H358 cells thereby leading to a p53- and Fas-independent pathway via mitochondria (Zhao et al., 2007). In laryngeal HEp-2 cells, *in vitro* and HEp-2 cell-transplanted BALB/c nude mice, *in vivo*, elemene enhanced the expression of caspase-3, thereby inhibiting the eukaryotic initiation factors (eIF4E and eIF4G), basic fibroblast growth factor (bFGF), and vascular endothelial growth factor (VEGF) (Tao et al., 2006). Collectively, the results of these studies elucidate that the antineoplastic effect of elemene in cancers of respiratory system is by the induction of apoptosis.

### Immune System

Five piperazine derivatives of β-elemene were synthesized and their antileukemic effect was tested against HL-60, NB4, K562m and HP100-1. DX-1 was effective in induction of apoptosis in HL-60 cell line by both receptor and mitochondria mediated pathways. Caspase-8 activation was correlated with the decrease in the levels of cellular FLICE-inhibitory protein (c-FLIP), an inhibitor of apoptosis triggered by engagement of death receptors (Yu et al., 2011). When treated against HL-60 cells, elemene enhanced the apoptotic effect induced by aclarubicin and down-regulated COX-2, NF-kappaB and PGE2 expressions (Zheng et al., 2009). When elemene was treated against human multiple myeloma cell line RPMI-8226, caspase-3, and DR-4 protein expressions increased, whereas, bcl-2, NF-kappaB and P65 protein expressions decreased. Apoptosis induced by elemene may be by activation of mitochondrial and death receptor mediated pathways, according to the authors (Chen et al., 2010).

An elemene derivative named N-(beta-elemene-13-yl) tryptophan, treated against human acute promyelocytic leukemia NB4 cells resulted in apoptotic cell death mediated by hydrogen peroxide generation and activation of caspase-3 (Yu, 2010). Taken together, elemene exerts its antineoplastic on cancers of immune system through induction of apoptosis.

### Digestive System

The copper transporter 1 levels are increased by treatment with elemene, which can allow the entry of oxaliplatin into hepatocellular carcinoma cell lines MHCC97H and Hep3B. This will result in increased sensitivity of cancer cells to oxaliplatin (Li et al., 2016). Against the hepatoma cell line HepG2, elemene can induce cell cycle arrest at G_2_/M phase and cell death by apoptosis by upregulating Fas/FasL expression (Dai et al., 2013). Multidrug resistant variant human gastric adenocarcinoma SGC7901/ADM cell line was treated with elemene. Apoptosis was observed with increasing concentrations of elemene and it also increased the sensitivity of the cell line to adriamycin which might be due to inhibition of NF-kappaB activity (Fu et al., 2013). Elemene suppressed the proliferation of esophageal carcinoma ECA-109 cells by inhibiting the human telomerase reverse transcriptase expression using a long noncoding RNA CDKN2B-AS1 (Hu et al., 2015). These results also stand supportive for the antineoplastic effect of elemene through an apoptotic trigger.

### Reproductive System

Elemene inhibited the proliferation of androgen-insensitive prostate carcinoma DU145 and PC-3 cells and induced apoptosis as observed by TUNEL assay and flow cytometric analysis. This was further evidenced by decreased levels of bcl-2, increased levels of cytochrome c release, activated PARP and caspase-3, -7, -9, and -10 in prostate cancer cells after elemene treatment (Li et al., 2010a). In a similar study, elemene treatment against the androgen-independent prostate carcinoma DU145 and PC-3 cells resulted in mitochondria mediated apoptosis. Elemene acted as a chemosensitizing agent for cisplatin in this study by augmenting the cisplatin-induced activation of caspases-3, 7, 9, and 10, cleavage of caspase-3 and -9, suppression of Bcl-2 and Bcl-XL expression, and release of cytochrome c from the mitochondria of these cells (Li et al., 2010b).

In the cisplatin-sensitive human ovarian cancer cell line A2780 and its cisplatin-resistant counterpart A2780/CP, treatment of elemene alone or in synergism with cisplatin, resulted in alterations in cyclin and cyclin-dependent kinase expression, including the down-regulation of CDC2, cyclin A, and cyclin B1, and the up-regulation of p21WAF1/CIP1 and p53 proteins caused cell cycle arrest at the G2/M phase. There was an apoptotic trigger which was an outcome of the activation of caspases-3, -8, and -9, the loss of mitochondrial membrane potential (ΔΨm), the release of cytochrome c into the cytosol and changes in the expression of bcl-2 family proteins (Li et al., 2005; Lee et al., 2012). Similar results were obtained by yet another study, with elemene inducing a potential apoptotic trigger (Lee et al., 2012). In the breast cancer MB-468 cells, elemene in synergism with paclitaxel can down-regulate the cell cycle protein cyclin-B1 expression and up-regulate the P27(kip1) expression thereby leading to an inhibition of cell proliferation (Cai et al., 2013). The human cisplatin-resistant ovarian cancer cell lines A2780/CP and MCAS were chemosensitized by elemene through the induction of apoptosis, mediated by a mitochondria- and caspase-dependent (caspase-3/8/9) cell death pathway. It upregulated the expression of pro-apoptotic Bax and downregulated the anti-apoptotic Bcl-2 and Bcl-XL expressions (Li et al., 2013c).

Therefore, the cancers of reproductive system are susceptible to elemene via an apoptotic trigger as observed through the outcomes of these experiments.

### Endocrine System

Synergistic interaction of rapamycin and elemene influenced a significant antiproliferative effect in FTC-133 thyroid cell line, dependent on inhibiting the AKT feedback activation induced by Rapamycin (Zhou et al., 2016). Inhibition of Akt activity by Rapamycin has been related to induction of apoptosis (Dormond et al., 2007). Therefore, the inhibition of Akt by Rapamycin in this study could be correlated to an apoptotic trigger.

### Urinary System

Elemene enhanced the activity of cisplatin by chemosensitization against human bladder cancer 5637 and T-24 cells by elevating expressions of caspase-3, -7, -8, -9, and -10 leading to apoptosis through a caspase dependent mechanism (Li et al., 2013e). Elemene downregulates the expression of survivin, Bcl-xL, Mta-1 and induces apoptosis in human bladder cancer T24 cells in a time and dose dependent manner (Chen et al., 2012). This suggests that apoptosis is the rationale behind the observed antineoplastic effects in cancers of urinary system.

## Sensitization of Multidrug Resistant Cancer Cells

Elemene increased the susceptibility of multidrug resistant cancer cells toward the respective drugs in several cases. It increased the sensitivity of MDR leukemia (K562/DNR) and gastric cancer lines (SGC7901/ADR), thereby enhancing the cytotoxic activity of P-gp substrates (DOX, DNR, and EPI). This was achieved by downregulation of Akt phosphorylation and upregulation of the E3 ubiquitin ligases, c-Cbl and Cbl-b (Zhang et al., 2013). In combination with anti-neoplastic drugs (colchicine, vinblastine and paclitaxel), elemene was reported to produce effective cytotoxicity by blocking efflux portion of ABCB1 transporters that are over-expressed in KB-C2 cells (Guo et al., 2014). In human BCA adriacin (Adr) – resistant MCF-7 cells (MCF-7/Adr) and docetaxel (Doc) – resistant MCF-7 cells (MCF-7/Doc), PTEN expression was significantly increased, whereas, Pgp expression was decreased after treatment with elemene (Zhang et al., 2014).

## Synergistic Anticancer Effects

In lung cancer cell lines NSCLC H460 and A549, elemene enhances cisplatin activity by increasing checkpoint kinase (CHK2) and reducing CDC2 (Li et al., 2013d). Elemene along with taxanes exhibited moderate synergistic antitumor activity against ovarian (A2780/CP70) and prostate (PC-3) carcinomas *in vitro* by increasing permeabilization of taxanes, micronuclear formation, and binding with efflux pumps along with upregulated expression of caspase-9 and p53 (Zou et al., 2013). It sensitized ovarian carcinoma cells to cisplatin treatment by down-regulating excision repair cross-complementation group-1 (ERCC-1) and XIAP through JNK pathway (Li et al., 2013a). It potentiated the activity of endostar, a recombinant human endostatin by inhibiting VEGF and MMP-2 leading to a decrease in malignant ascites formation in H22 murine model (Jiang et al., 2012).

## Contradictory Effects of Elemene

In human gastric cancer cell lines MGC803 and SGC7901, protective autophagy was observed. According to the authors, the combination of autophagy inhibitors such as 3-methyladenine (3-MA) or chlorochine with elemene can induce apoptosis in the cancer cells by causing an increase in antitumor effects of elemene. Contraindicative to anticancer effects of elemene, when treated alone, it induced protective effects on gastric cancer cells (Liu et al., 2011). Similar observations of protective autophagy were attained against the breast cancer Bcap37 and MBA-MD-231 cells (Guan et al., 2014).

## Protective Effect Against Normal Cells

Elemene exhibited low toxicity toward lung fibroblast (CCD-19Lu) and human bronchial epithelial (NL20) normal cell lines (Wang et al., 2005). In addition, elemene showed less toxicity to ovarian epithelial cell line (IOSE-397) (Li et al., 2005). It showed limited toxicity to macrophages and reduced infiltration of the same in rabbit *in vivo* model (Zhong et al., 2015).

## Future Directions

Mechanistic approaches for the treatment of cancer, the modern epidemic, using elemene had made considerable progress over the recent past, resulting in decreased mortality rate. Simultaneously, the disease susceptibility and drug responses for elemene can be identified using specific molecular approaches. The studies of this kind are still at their infancy. Although available reports have cited elemene to be minimally toxic, this effect can be elucidated better. Further, elemene-induced liver injury has to be monitored. Adding to this, nano-formulations of elemene can be derived to enhance the bioavailability and efficacy of elemene, such that of nano-curcumin. This will eventually lead to a better, more specific and efficient anticancer therapy, devoid of side effects. Future studies that can elicit the exact role of elemene in anticancer therapy and novel, specific drug development methods are the need of the hour.

## Conclusion

**FIGURE 1:**
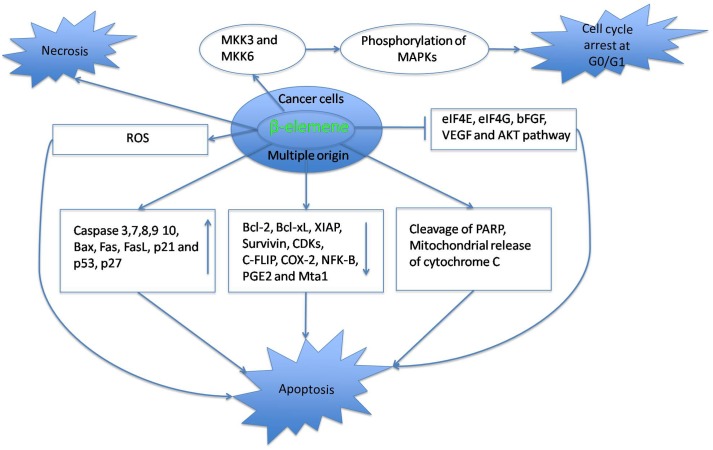
**Modes of cell death induced by β-elemene**.

Elemene has been proven to possess anticancer activity. Decrease in mitochondrial potential, upregulation of pro-apoptotic signals and downregulation of anti-apoptotic signals are predominantly observed in cancer cells treated with elemene. Therefore, from the above results, it can be suggested that apoptosis has led to the anticancer activity of elemene (**Table 1** and **Figure 1**). This is consistent with the molecular mechanism studied for other preclinical and clinical drugs. The mini-review based on the information presented, concludes that the β-elemene can be a precious candidate for future anticancer medications.

**Table 1:** Anticancer effect of β-elemene against cancer cell lines of multiple origin.

S. no	Cell line type	Genes/proteins involved	Mode of action	Reference
**Integumentary system**		
1.	Melanoma cells (B16F10), Melanoma mice (C57BL/6)	VEGF	Antiangiogenesis	Chen et al., 2011
**Nervous system**		
2.	Glioblastoma cell line (U87)	MKK3 and MKK6	Cell cycle arrest G0/G1 phase	Zhu et al., 2011b
3.	Glioblastoma cell line (U87)	Glia maturation factor β	ERK1/2-Bcl-2/survivin pathway leading to apoptosis	Zhu et al., 2014
4.	Glioblastoma cell line (C6 and U251)	p38 MAPK phosphorylation	Cell-cycle arrest at the G0/G1phase	Yao et al., 2008a,b
5.	Brain tumor cell lines (A172, CCF-STTG1, and U-87MG)	Caspase-3/7/10, Bax, Bcl-2, Bcl-XL, and XIAP	Apoptosis	Li et al., 2013a
6.	Glioma cell lines (U251 and A172)	Caspase 9/3/7 and survivin	Apoptosis	Zhang et al., 2012
7.	Glioma cell lines (C6 and SHG-44)	Apoptotic bodies	Apoptosis	Zhou et al., 2003
**Respiratory system**		
8.	NSCLC cell lines (H460 and A549)	Caspase 3/7/9, Bcl-2, and cytochrome C	Apoptosis	Wang et al., 2005
9.	NSCLC cell line (A549)	Bax, p53, p21, PARP, and cyclin D1.	Apoptosis	
10.	NSCLC cell lines (PC9 and H1299)	p21	Apoptosis	Zhao et al., 2011
11.	NSCLC cell lines (H460 and A549)	Bax, phospho-Bcl-2, Bcl-2, and XIAP.	Apoptosis	Li et al., 2009
12.	NSCLC cell lines (PC9, H1299, H1650, A549, H358 and H1975)	ERK1/2- and AMPKα	Apoptosis	Zhao et al., 2015
13.	NSCLC cell lines (p53 mutant H23 cells and p53 null H358 cells)	Bcl-2, Bcl-XL, caspase-8/3	Apoptosis	Zhao et al., 2007
14.	Laryngeal cell line (HEp-2), HEp-2 cell-transplanted BALB/c nude mice	Caspase-3, eIF4E, eIF4G, bFGF and VEGF	Apoptosis and angiogenesis	Tao et al., 2006
**Immune system**		
15.	Leukemia cell line (HL-60)	Caspase-8, c-FLIP	Apoptosis	Yu et al., 2011
16.	Leukemia cell line (HL-60)	COX-2, NF-kappaB, and PGE2	Apoptosis	Zheng et al., 2009
17.	Multiple Myeloma cell line RPMI-8226	Caspase-3 and DR-4, bcl-2, NF-kappaB, and p65	Apoptosis	Chen et al., 2010
18.	Acute promyelocytic leukemia cell line (NB4)	Caspase-3	ROS, Apoptosis	Yu, 2010
**Digestive system**		
19.	Hepatocellular carcinoma cell lines (MHCC97H and Hep3B)	Fas/FasL	Cell cycle arrest (G_2_/M) and apoptosis	Dai et al., 2013
20.	Gastric adenocarcinoma cell line (SGC7901/ADM)	NF-kappaB	Apoptosis	Fu et al., 2013
**Reproductive system**		
21.	Prostate carcinoma cell line (DU145 and PC-3)	Bcl-2, Activated PARP, caspase 3/7/9/10, and cytochrome c	Apoptosis	Li et al., 2010a
22.	Prostate carcinoma cell line (DU145 and PC-3)	Caspases-3/7/9/10, Bcl-2, and Bcl-XL	Apoptosis	Li et al., 2010b
23.	Cisplatin-sensitive human ovarian cancer cell line (A2780)	CDK CDC2, cyclin A, cyclin B1, p21WAF1/CIP1, p53, caspases 3/8/9	Cell cycle arrest at the G2/M phase	Li et al., 2005; Lee et al., 2012
24.	Breast cancer cell line (MB-468)	Cyclin-B1 expression and up-regulate the p27	Cell cycle inhibition	Cai et al., 2013
25.	Cisplatin-resistant ovarian cancer cell lines (A2780/CP and MCAS)	Caspase-dependent cell death	Apoptosis	Li et al., 2013b
**Endocrine system**		
26.	Thyroid cell line (FTC-133)	AKT feedback activation	Apoptosis	Zhou et al., 2016
27.	Bladder cancer cell line (5637 and T-24)	Caspase 3/7/8/9/10	Apoptosis	Li et al., 2013c
28.	Bladder cancer cell line (T24)	Bcl-xL, Mta-1, and survivin	Apoptosis	Chen et al., 2012

## Author Contributions

ZJ wrote the article; JJ edited the article; DL and PN revised the article; BC designed and approved the submission.

## Conflict of Interest Statement

The authors declare that the research was conducted in the absence of any commercial or financial relationships that could be construed as a potential conflict of interest.
